# Childhood cumulative risk and relationship addiction: a developmental timing-pathway differentiation-node buffering integrative model

**DOI:** 10.3389/fpsyg.2026.1839101

**Published:** 2026-06-24

**Authors:** Siyao Yan, Ruiqian Li, Senwei Fang

**Affiliations:** Department of Early Childhood Education, School of Education and Psychology, Shaoxing University, Shaoxing, China

**Keywords:** childhood cumulative risk, developmental timing, DT-PD-NB model, parental attachment, relationship addiction, resilience

## Abstract

**Introduction:**

To challenge the traditional view of homogeneous effects of childhood risk, this study proposed and preliminarily examined an integrative “Developmental Timing-Pathway Differentiation-Node Buffering” (DT-PD-NB) model to explore the heterogeneous association pathways between childhood cumulative risk and adult relationship addiction.

**Methods:**

This study adopted a cross-sectional retrospective survey design, and a questionnaire survey was conducted among 856 Chinese adults. Structural equation modeling was employed with cross-validation, *E*-value sensitivity analysis, systematic competitive model comparison, and placebo testing to enhance causal inference credibility.

**Results:**

The results identified three potential association patterns: (1) Temporal specificity: Infancy risk primarily showed indirect effects through attachment security impairment (*β*_indirect_ = .21, 95% CI [0.16, 0.27]), whereas late school-age risk demonstrated strong direct effects (*β*_direct_ = .34, 95% CI [0.30, 0.38]); (2) Pathway differentiation: The maternal attachment mediation pathway (*β*_indirect_ = .13, 95% CI [0.09, 0.18]) was twice as strong as the paternal pathway (.06, 95% CI [0.03, 0.10]), with stronger effects among females; (3) Node buffering: Ego-resiliency buffered risk damage to attachment (interaction *β* = −.18, *p* < .001), while family routines blocked risk erosion of psychological resilience (interaction *β* = −.22, *p* < .01).

**Discussion:**

The childhood risk effects follow refined, adjustable developmental pathways. This provides a novel framework for understanding the origins of relationship addiction and designing precision prevention strategies tailored by developmental stage, target population, and intervention node. This study employed a cross-sectional retrospective design; all findings represent associational evidence awaiting confirmation through prospective longitudinal research.

## 1 Introduction

### 1.1 Conceptual definition and theoretical boundaries of relationship addiction

Despite growing research interest in relationship addiction, conceptual ambiguity and overlapping constructs remain significant barriers to theoretical advancement and clinical practice (Redcay and Simonetti, 2018; Cavalli et al., 2025). To address this gap, this section explicitly defines the core characteristics of relationship addiction and clarifies its theoretical boundaries from two closely related but distinct constructs: attachment anxiety and interpersonal dependency.

- Five core characteristics of relationship addiction. Consistent with contemporary behavioral addiction frameworks (Sussman et al., 2011), relationship addiction is defined as a persistent, maladaptive pattern of extreme emotional and behavioral dependence on intimate relationships that causes significant impairment in multiple domains of functioning. This construct exhibits five interrelated core characteristics: (1) Excessive emotional dependency: An overwhelming need for constant validation, reassurance, and proximity from a romantic partner, with self-worth entirely contingent on the relationship’s status (Redcay and McMahon, 2021); (2) Pathological jealousy and possessiveness: Irrational fears of infidelity, intrusive monitoring of the partner’s activities, and attempts to control the partner’s social interactions (Schimmenti and Caretti, 2016); (3) Separation anxiety and abandonment panic: Severe distress, panic attacks, or depressive symptoms when separated from the partner, even for short periods (Willoughby and Dover, 2024); (4) Emotion dysregulation: Inability to modulate emotions in response to relationship stressors, leading to impulsive behaviors, self-harm, or aggressive outbursts (Cavalli et al., 2025); (5) Compulsive relationship maintenance: Persistent efforts to sustain dysfunctional or abusive relationships despite negative consequences, including loss of personal identity, social isolation, or physical harm (Costa et al., 2021).
- Distinction from attachment anxiety. While relationship addiction and attachment anxiety are positively correlated (meta-analytic *r* = 0.56; Cavalli et al., 2025), they represent theoretically distinct constructs. Attachment anxiety is a stable personality trait rooted in early insecure attachment experiences, characterized by a generalized fear of abandonment and negative self-views across all relational contexts (Mikulincer and Shaver, 2007). In contrast, relationship addiction is a specific behavioral addiction pattern that manifests primarily in intimate romantic relationships, with core features of compulsion, loss of control, and functional impairment that are not inherent to attachment anxiety. The moderate correlation observed between the two constructs suggests that attachment anxiety may serve as a vulnerability factor for relationship addiction, but it is not synonymous with the disorder (Redcay and McMahon, 2021).
- Distinction from interpersonal dependency. Relationship addiction also differs from interpersonal dependency, a broader personality trait defined as a pervasive need to be taken care of by others across all social and professional domains (Willoughby and Dover, 2024). Interpersonal dependency is characterized by submissiveness, difficulty making independent decisions, and fear of disapproval in all relationships, whereas relationship addiction is specifically directed toward a single romantic partner and exhibits the hallmark features of addiction, including tolerance (increasing need for proximity over time), withdrawal symptoms (distress when separated), and repeated failed attempts to reduce dependency (Costa et al., 2021). This distinction is critical for clinical practice, as interventions targeting general interpersonal dependency may not address the specific addictive processes underlying relationship addiction.

By clarifying these conceptual boundaries, this study provides a precise operational definition of relationship addiction that guides subsequent measurement and analysis, ensuring that findings are comparable to existing literature and directly relevant to clinical intervention.

### 1.2 The current state of research on childhood cumulative risk and relationship addiction

Relationship addiction, characterized by excessive emotional dependency, pathological jealousy, and separation anxiety in intimate relationships (Redcay and McMahon, 2021; Kardefelt-Winther et al., 2017; Schimmenti and Caretti, 2016), represents an emerging behavioral addiction subtype of increasing clinical and research attention (Sussman et al., 2011; Cavalli et al., 2025). Core manifestations include pathological relational dependency, emotion dysregulation, and self-boundary dissociation (Willoughby and Dover, 2024). Accumulating evidence consistently identifies early childhood experiences as critical determinants of adult intimate relationship patterns (Hays-Grudo et al., 2021; Wilke et al., 2020). However, substantial theoretical gaps persist regarding the specific developmental processes through which childhood cumulative risk is associated with relationship addiction outcomes (Redcay and Simonetti, 2018).

Attachment theory provides a foundational framework for understanding these early influences. Bowlby’s (1982, 1988) internal working model theory posits that early parent–child interactions form enduring affective and cognitive schemas that profoundly shape adult relational behavior. Hazan and Shaver (1987), subsequently elaborated by Mikulincer and Shaver (2007) and Simpson (1990), further described the associations between insecure attachment and relationship anxiety or dysfunction. Nevertheless, classical attachment theory exhibits three critical limitations when applied to relationship addiction development that warrant theoretical integration.

First, existing research has not yet fully explored the “developmental stage specificity” mechanism of childhood risk effects (Schaefer et al., 2022; Gabard-Durnam and McLaughlin, 2020). Although Bronfenbrenner’s (1994) bioecological theory emphasizes developmental timing, current models inadequately reveal the differential effects of risk exposure across distinct childhood periods. Infancy, as a sensitive period for attachment formation, may impair secure base development and undermine the neurobiological foundations of emotion regulation (Thompson, 2013; Gunnar, 2020), whereas late school-age, as a critical period for social-cognitive development, may more directly disrupt relationship-behavior regulatory systems (Moutsiana et al., 2014; Guyer et al., 2016). This theoretical distinction regarding differential risk effects across developmental stages is essential for understanding addiction pathway origins and enabling precision intervention (Andersen, 2015; Barzilay, 2024), constituting the first critical theoretical gap.

Second, the differential contributions of maternal versus paternal attachment pathways require further theoretical clarification (Hoenicka et al., 2022; Lee, 2024). Although Main’s differentiated attachment theory proposes parental role specialization—mothers primarily providing emotional nurturing while fathers support exploration (Bretherton, 2010; Lamb and Tamis-LeMonda, 2004)—no comprehensive model has systematically articulated how these differentiated pathways independently mediate adult relationship addiction. Particularly within East Asian cultural contexts, Rothbaum’s attachment reciprocity theory emphasizes maternal dominance in offspring emotional socialization through affective co-regulation (Rothbaum et al., 2007; Joo et al., 2025), while cross-cultural neuroscience evidence reveals adaptive reorganization of prefrontal-limbic system functioning (Chiao et al., 2009; Kitayama and Park, 2010; Ma et al., 2020). This suggests potential culture-specific advantages for maternal attachment pathways. The differentiation of parental attachment pathways and their operation within specific cultural contexts constitutes the second core theoretical gap.

Third, theoretical explanations regarding protective factor regulatory pathways require further development (Masten, 2014; Abate et al., 2024). Although Masten’s (2014) resilience framework identifies individual psychological resilience as a core mediator, it does not fully clarify how different protective factors function at distinct nodes within risk transmission pathways. Block’s ego-resiliency theory proposes that trait-like regulatory capacity may buffer risk effects on attachment representations (Block and Kremen, 1996; Galatzer-Levy et al., 2018; Dunkel et al., 2021), whereas Boyce’s (2015) family routine stability theory suggests that environmental structural factors are more likely to block negative risk-to-resilience transformation (Hosokawa et al., 2023; Keane and Shelleby, 2024). The absence of node-specific protective factor models constrains the development of multi-level protection strategies (Ungar, 2015; Van Schoors et al., 2023), representing the third major theoretical gap.

However, the theoretical advances described above remain governed by a more fundamental paradigm—the cumulative risk model (Stein et al., 2022; Doom et al., 2020). While this model is heuristic, its core assumption of homogeneity (that risks erode development in an additive, equivalent manner) cannot fully account for why identical risk “doses” lead to heterogeneous outcomes. Specifically, does the impact of risk vary depending on developmental timing (When)? Does it transmit through differentiated relational pathways (Through Whom)? And at which nodes can it be specifically buffered (Where to Protect)?

**Figure 1:**
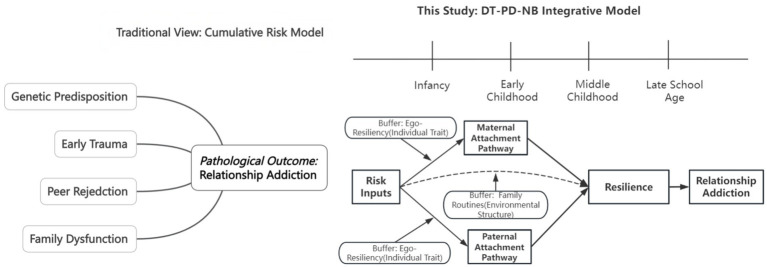
The Developmental Timing-Pathway Differentiation-Node Buffering (DT-PD-NB) integrative model.

To directly address the homogeneity assumption and systematically address these questions, this study proposes the “Developmental Timing-Pathway Differentiation-Node Buffering” (DT-PD-NB) Integrative Model (see Figure 1). This model represents a paradigmatic perspective shift: from calculating risk “totals” to dissecting the “heterogeneous pathway system” of risk transmission. We hypothesize that the operation of this system is associated with whether and how childhood risk connects to adult relationship addiction.

Based on this model, we propose the following specific hypotheses:

*H1 (Temporal Specificity Hypothesis)*: Infancy risk would be expected to primarily show indirect effects on relationship addiction through attachment security impairment, whereas late school-age risk would be expected to demonstrate stronger direct effects.

*H2 (Pathway Differentiation Hypothesis)*: Maternal attachment would be expected to show stronger mediation effects than paternal attachment, with this difference being more pronounced among females.

*H3 (Node-Specific Buffering Hypothesis)*: Ego-resiliency would be expected to specifically moderate the Childhood Risk to Parental Attachment pathway, while family routine stability will specifically moderate the Childhood Risk to Resilience pathway.

*H4 (Cumulative Effect Hypothesis)*: The chain mediation pathway (Childhood Risk to Parental Attachment to Resilience to Relationship Addiction) would be expected to remain significant after controlling for current emotional states and alternative model specifications.

## 2 Method

### 2.1 Participants and procedure

This study employed a cross-sectional retrospective survey design conducted from March to May 2025 through a professional online research platform. Participants were adult residents aged 18–45 years from a coastal province in Eastern China. A stratified random sampling strategy was implemented, combining geofencing technology with mobile population behavior compensation strategies to achieve sample representativeness across gender, age, education, income, and urban–rural distribution. To mitigate retrospective reporting bias, this study employed four strategies: (1) separating childhood measurement modules from adult trait modules in questionnaire presentation; (2) using multi-dimensional risk indicators across 8 categories and 4 developmental stages; (3) converting risk scores to percentiles for cross-stage comparison; and (4) statistically controlling for current depression and anxiety symptoms in all analyses. Nevertheless, retrospective self-report methods cannot completely eliminate memory reconstruction effects, particularly for infancy period (0–2 years) recollections.

A total of 1,000 questionnaires were distributed. Prior to data collection, ethical approval was obtained from the Ethics Committee of the School of Education and Psychology, Shaoxing University. Questionnaires included three attention-check items and monitored response times (valid range: 8–60 min). Module order proceeded as: demographic information, childhood retrospective measures, and adult trait measures, with average completion time of approximately 22.6 min. Following platform quality control and manual verification, 856 valid questionnaires were obtained (effective response rate: 85.6%).

Initial sample demographics deviated from provincial census data. Therefore, three-stage post-stratification weighting was applied: (1) Base weights based on age-stratified structure across 11 prefecture-level cities (range: 1.05–1.71); (2) Compensation weights for economically developed counties with high mobile populations (37.0%), applying additional weights for economic strong counties (x1.82) stratified by residence duration and origin; (3) Minimum sample guarantee ensuring ≥50 participants per mountain county. Following weighting, sample key demographic characteristics showed no significant differences from provincial statistics (all *p* > 0.05), indicating good representativeness.

Weighted sample characteristics were: Male 47.0%, Female 53.0%; Age distribution: 18–25 years (26.3%), 26–35 years (38.1%), 36–45 years (35.6%); Education: High school or below (52.3%), College (27.2%), Bachelor’s or above (20.5%); Family income: <50,000 RMB (16.8%), 50,000–150,000 RMB (52.7%), >150,000 RMB (30.5%); Residence: Central city (31.8%), Coastal industrial city (28.4%), Mountain/underdeveloped county (16.2%), Other counties/towns (23.6%).

### 2.2 Measures

All measures employed Likert-type scoring, with good reliability demonstrated in the current sample.

#### 2.2.1 Relationship addiction tendencies

The Relationship Addiction Questionnaire (RAQ-30; Woo, 2014; Costa et al., 2021) assessed adult relationship addiction tendencies. This 30-item unidimensional measure assesses core symptoms including excessive dependence, relationship maintenance difficulties, and pathological jealousy. Items were rated on a 5-point scale (1 = Strongly Disagree to 5 = Strongly Agree), with higher total scores indicating greater addiction tendency. Cronbach’s alpha = 0.942 in this study. Confirmatory factor analysis supported good structural validity (CFI = 0.961, RMSEA = 0.052, SRMR = 0.048). Discriminant validity analysis showed that RAQ-30 scores correlated with attachment anxiety scale scores at *r* = 0.58 (*p* < 0.001) and with interpersonal dependency scale scores at *r* = 0.52 (*p* < 0.001), supporting the construct independence of relationship addiction. Confirmatory factor analysis supported the unidimensional structure of RAQ-30 (χ^2^/df = 1.88, CFI = 0.961, RMSEA = 0.052, SRMR = 0.048).

#### 2.2.2 Parental attachment quality

The Inventory of Parent and Peer Attachment-Revised (IPPA-R; Armsden and Greenberg, 1987; Hoenicka et al., 2022) father and mother subscales assessed retrospective evaluations of childhood parental attachment security. Each subscale contains 24 items across three dimensions: Trust, Communication, and Alienation. Items were rated on a 5-point scale, with higher scores indicating greater perceived attachment security. Cronbach’s alpha = 0.927 for paternal attachment and alpha = 0.901 for maternal attachment.

#### 2.2.3 Psychological resilience

The Resilience Quotient Test (RQT; Shin et al., 2009; Song et al., 2021) assessed current resilience levels. This 27-item measure includes three dimensions: Control, Positivity, and Sociality. Items were rated on a 5-point scale (total score range: 27–135), with higher scores indicating greater resilience. Cronbach’s alpha = 0.931 in this study.

#### 2.2.4 Childhood retrospective variables

Childhood ego-resiliency was assessed using Song’s (2025) revised version of Block and Kremen’s (1996) Ego-Resiliency Scale, comprising 14 items rated on a 4-point scale (higher mean scores indicating better childhood ego-resiliency; Dunkel et al., 2021; Pyszkowska, 2020). Cronbach’s alpha = 0.812. Childhood family routine stability was measured using a revised version of Sytsma et al.’s (2001) scale, with 5 items assessing maternal consistency in establishing rules and routines during childhood, rated on a 5-point scale (Romano et al., 2022; Hosokawa et al., 2023). Cronbach’s alpha = 0.791.

This study explicitly distinguishes between two resilience constructs at different developmental levels. Childhood ego-resiliency (Block and Kremen, 1996) is a stable personality trait formed in early childhood, referring to the capacity to flexibly regulate emotions and behaviors under stress and recover quickly from adversity. Because this trait is largely formed by early childhood, it can buffer the effects of risk exposure on parent–child interaction quality and attachment formation. Therefore, ego-resiliency is modeled as a MODERATOR on the Childhood Risk to Parental Attachment pathway. In contrast, adult psychological resilience (Shin et al., 2009) is a broader developmental construct encompassing control, positivity, and sociality dimensions. It develops gradually on the foundation of secure attachment relationships and protects individuals from negative life events. Therefore, psychological resilience is modeled as a MEDIATOR connecting parental attachment to relationship addiction. This distinction is supported by prior research showing that childhood ego-resiliency is an important antecedent of adult psychological resilience, and the two constructs are theoretically and empirically distinct (Dunkel et al., 2021).

#### 2.2.5 Childhood cumulative risk index

Following Song (2025) and Stein et al. (2022), participants retrospectively reported exposure to 8 categories of risk factors across four developmental stages (infancy: 0–2 years, toddlerhood: 2–4 years, preschool: 4–6 years, late school-age: 6–12 years). The 8 risk categories included: (1) emotional neglect, (2) physical neglect, (3) emotional abuse, (4) physical abuse, (5) sexual abuse, (6) parental divorce/separation, (7) family poverty, and (8) parental substance abuse. Each stage’s risk score was the sum of exposed risk factors, converted to percentile ranks for cross-stage comparison. KR-20 reliability coefficients ranged from 0.74 to 0.81 across developmental stages.

### 2.3 Causal inference credibility enhancement and robustness analysis strategy

**Figure 2:**
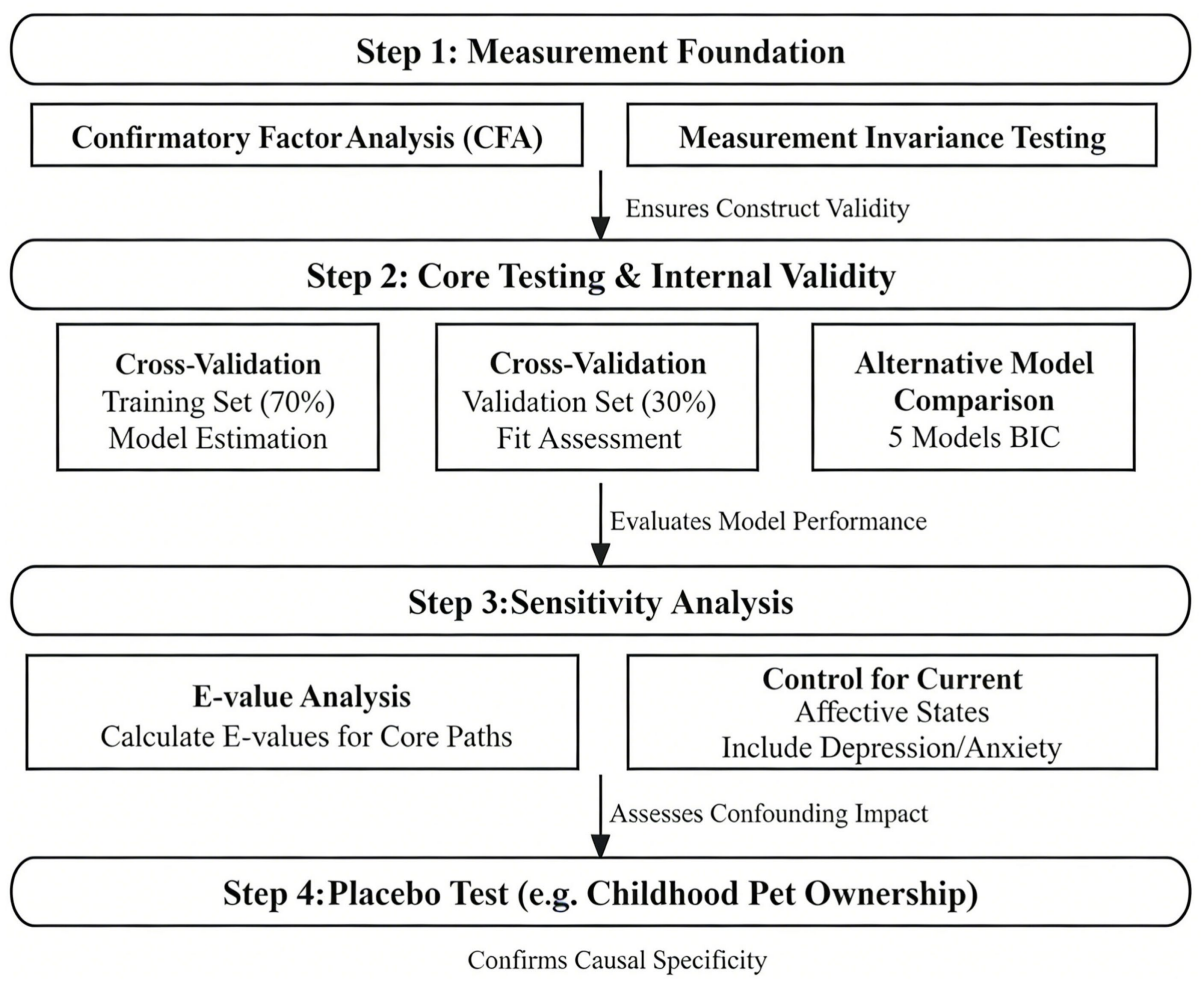
Causal inference credibility enhancement strategy framework.

This study employed a cross-sectional retrospective design. We fully recognize that any path coefficients derived from such data face threats from multiple competing alternative explanations (e.g., common method bias, reverse causality, current state contamination of recall; Savitz and Wellenius, 2023; Imai et al., 2023). To actively assess and minimize these threats, we did not conduct a single analysis but executed a progressive “Causal Inference Credibility Enhancement Strategy” (see Figure 2). Each step of this ladder aims to exclude a specific category of alternative explanations, thereby accumulating incremental evidential weight for the core argument that “the DT-PD-NB model depicts potential mechanisms” (Zhao and Sun, 2024).

#### 2.3.1 Step 1: measurement model and baseline associations

Using Mplus 8.3 with robust weighted least squares for confirmatory factor analysis, we reported composite reliability (CR), average variance extracted (AVE), and conducted strong measurement invariance tests across gender and age groups (Andre et al., 2020).

#### 2.3.2 Step 2: Core model testing and internal validity

- Sample splitting and cross-validation: Using set.seed(123) in R, the total sample was randomly divided into training (70%) and validation (30%) sets. Structural equation models were estimated on the training set using type = “complex.” Subsequently, all parameters (factor loadings, path coefficients, intercepts) from the training set were fixed, and model fit indices (CFI, RMSEA, SRMR, TLI) were calculated on the validation set to assess model generalizability.
- Systematic competitive model comparison: We fitted and compared five models: (1) Theoretical model (M1); (2) Common method factor model (M2); (3) Reverse causation model (M3, predicting attachment and resilience from relationship addiction); (4) No moderation model (M4); (5) No parental pathway differentiation model (M5, constraining maternal and paternal path coefficients to be equal). Bayesian Information Criterion (BIC) was used for model selection, with ΔBIC reported. BIC differences greater than 10 were considered strong evidence supporting the model with lower BIC.

#### 2.3.3 Step 3: sensitivity analysis

- *E*-value analysis: To quantify the robustness of core findings against “unmeasured confounding,” we calculated *E*-values for all direct, indirect, and moderation effects. This metric answers the question: “How strong would an unmeasured confounder (e.g., a specific gene or unrecorded trauma) need to be to completely overturn the observed association?
- Controlling for current emotional states: To directly test the most compelling alternative explanation—that “current relationship distress distorts childhood recall”—we included current depression/anxiety symptoms as covariates in the model. If core paths remain robust after this control, the persuasiveness of the “reverse causality” explanation is significantly weakened.

#### 2.3.4 Step 4: specificity test (placebo test)

We selected “number of siblings” as a placebo variable. After standardization, it was substituted into the exact same latent moderated structural equation model as “ego-resiliency” to test its interaction with childhood risk in predicting maternal attachment. We expected this interaction to be non-significant (*p* > 0.10).

### 2.4 Theoretical construction logic and parameter justification of the DT-PD-NB model

The integrative model proposed in this study, although containing multi-dimensional variables and paths, is not a data-driven exploratory model but a systematic, hierarchical integration based on three classical theories. Every path and every moderation effect has explicit *a priori* theoretical basis, with no *post-hoc* exploratory paths added. The model construction follows a strict logical chain of risk input to mediation transmission to moderation buffering to outcome output.

First, theoretical basis sufficiency. The developmental timing subsystem is grounded in Bronfenbrenner’s (1994) bioecological theory and Andersen’s (2015) sensitive period hypothesis, examining differential effects of risk across four developmental stages. The pathway differentiation subsystem is based on Main’s differentiated attachment theory and Rothbaum et al.’s (2007) East Asian cultural attachment theory, distinguishing independent maternal and paternal mediation paths. The node buffering subsystem draws on Block’s ego-resiliency theory and Boyce’s (2015) family environment structure theory, testing protective factor specificity at different pathway nodes.

Second, sample size and parameter matching. This study has an effective sample size of 856 participants. The complete model contains 62 free parameters (47 measurement model + 15 structural model), yielding a N:parameter ratio of 13.8:1, substantially exceeding the recommended 10:1 threshold for structural equation modeling (Kline, 2016). *Post-hoc* statistical power analysis (alpha = 0.05, two-tailed) showed that for medium effect sizes (β = 0.20), the study achieves 0.97 power, stably detecting all theoretically expected effects.

Third, model parsimony versus explanatory power balance. Nested model comparisons (Section 3.2) verified the necessity of each added component. Compared to the baseline model containing only direct effects, the full model’s explanatory power (R^2^) for relationship addiction increased from 18.3 to 47.2%, with each step of model fit improvement reaching statistical significance (all Δχ^2^/df, *p* < 0.001), indicating that increased model complexity brings substantive explanatory power gains rather than overfitting.

## 3 Results

### 3.1 Measurement model validation

Following data weighting, measurement model validation was conducted. Multi-group confirmatory factor analysis demonstrated good construct validity and measurement invariance across genders. Configural invariance model fit: χ^2^/df = 1.92, CFI = 0.976, TLI = 0.969, RMSEA = 0.047, SRMR = 0.042. Strong invariance also satisfied equivalence criteria (ΔCFI = 0.007, ΔRMSEA = 0.003, ΔSRMR = 0.008), supporting subsequent cross-group comparisons. Full-sample measurement model demonstrated good fit (χ^2^/df = 1.88, CFI = 0.981, TLI = 0.975, RMSEA = 0.045, SRMR = 0.039), with all standardized factor loadings > 0.65 (*p* < 0.001). Common method bias assessment indicated method factor variance explanation (11.2%) below critical thresholds, suggesting minimal common method bias impact. Common method bias was assessed using the latent method factor approach recommended by Podsakoff et al. (2003), where all items were simultaneously loaded onto their theoretical factors and a common method factor. Results showed that the common method factor explained 11.2% of total variance, below the 20% critical threshold, indicating that common method bias in this study was within acceptable limits.

Composite reliability (CR) ranged from 0.78 to 0.92 across constructs, and average variance extracted (AVE) ranged from 0.51 to 0.68, indicating adequate convergent validity. Discriminant validity was supported as the square root of AVE for each construct exceeded its correlations with other constructs.

In addition to the overall measurement model, confirmatory factor analysis was conducted separately for each scale. Results showed satisfactory fit for all six scales: Relationship Addiction Questionnaire (RAQ-30): χ^2^/df = 1.88, CFI = 0.961, TLI = 0.954, RMSEA = 0.052 (90% CI [0.038, 0.065]), SRMR = 0.048, all standardized factor loadings > 0.62 (*p* < 0.001); IPPA-R Maternal subscale: χ^2^/df = 2.01, CFI = 0.958, TLI = 0.951, RMSEA = 0.054 (90% CI [0.041, 0.067]), SRMR = 0.046, all loadings > 0.60 (*p* < 0.001); IPPA-R Paternal subscale: χ^2^/df = 1.97, CFI = 0.962, TLI = 0.955, RMSEA = 0.052 (90% CI [0.039, 0.064]), SRMR = 0.043, all loadings > 0.58 (*p* < 0.001); RQT: χ^2^/df = 1.89, CFI = 0.972, TLI = 0.967, RMSEA = 0.046 (90% CI [0.033, 0.058]), SRMR = 0.038, all loadings > 0.62 (*p* < 0.001); Ego-Resiliency: χ^2^/df = 2.13, CFI = 0.947, TLI = 0.938, RMSEA = 0.059 (90% CI [0.046, 0.072]), SRMR = 0.051, all loadings > 0.55 (*p* < 0.001); Family Routines: χ^2^/df = 1.76, CFI = 0.968, TLI = 0.961, RMSEA = 0.043 (90% CI [0.030, 0.055]), SRMR = 0.040, all loadings > 0.61 (*p* < 0.001).

### 3.2 Nested model comparison and full model selection

To verify the superiority of the complete DT-PD-NB model, we constructed and compared six nested models, progressively adding theoretical components from the simplest baseline to the full model. Results (Table 1) showed that the full model (M5) achieved excellent fit with the lowest BIC. Each expansion significantly improved fit (all ΔBIC > 10; Raftery, 1995): adding dual parental attachment mediation improved by ΔBIC = 453.3; adding psychological resilience chain mediation by ΔBIC = 179.5; adding developmental stage differentiation by ΔBIC = 122.3; adding node-specific moderation by ΔBIC = 61.2; adding gender multi-group analysis by ΔBIC = 20.9. These results demonstrate that each DT-PD-NB component (developmental timing, pathway differentiation, node buffering) makes a unique and irreplaceable contribution.

**Table 1:** Nested model fit index comparison.

Model	Description	χ^2^/df	CFI	TLI	RMSEA	BIC	ΔBIC	*R* ^2^
M0 (baseline)	Risk to addiction direct effect only	4.21	0.823	0.796	0.094	9126.5	–	0.183
M1	+ Dual parental attachment mediation	2.76	0.912	0.897	0.064	8673.2	453.3	0.315
M2	+ Psychological resilience chain mediation	2.35	0.941	0.928	0.053	8451.7	179.5	0.389
M3	+ Developmental stage differentiation	2.12	0.958	0.947	0.048	8329.4	122.3	0.426
M4	+ Node-specific moderation	1.95	0.972	0.963	0.043	8268.1	61.2	0.458
M5 (full)	+ Gender multi-group analysis	1.88	0.981	0.975	0.045	8247.3	20.9	0.472

All ΔBIC values compared to the preceding model exceeded the strong evidence threshold of 10 (Raftery, 1995).

### 3.3 Robustness test results

First, we report a series of robustness test results (summarized in Table 2) to provide a credibility foundation for subsequent main findings.

**Table 2:** Summary of robustness test results.

Test type	Key indicator/result	Supporting conclusion
Common method bias	Latent method factor explained variance = 11.2%	CMV within acceptable range
Cross-validation	Validation set CFI = 0.962, RMSEA = 0.053, SRMR = 0.051	Good model generalizability
Competitive model comparison	M1 BIC = 8247.3 vs. M3 (Reverse causation, BIC = 8360.0), ΔBIC = 112.7 > 10	Theoretical model significantly superior
*E*-value analysis	Core path “Infancy risk to Maternal attachment to Resilience” indirect effect *E*-value = 2.47 [1.98, 3.12]	Results robust to unmeasured confounding
Controlling for current emotions	After controlling depression/anxiety, “Risk to Maternal attachment” path β change < 0.03	Path robust, not easily explained by current emotional states
Placebo test	“Sibling number x Risk” interaction effect on maternal attachment β = 0.03, *p* = 0.42	Ego-resiliency moderation effect has theoretical specificity
Risk trajectory classification	Four trajectory classes identified (BIC = 812.3, Entropy = 0.89)	Risk accumulation patterns show meaningful heterogeneity

Additionally, to address potential concerns regarding model complexity and overfitting, three supplementary robustness tests were conducted. First, leave-one-out cross-validation: the complete model was iteratively estimated with one participant removed at a time (N = 856 iterations). The average cross-validation R^2^ was 0.451, with a standard deviation of 0.023, indicating minimal difference from the original model R^2^ (0.472). This suggests the model shows no substantial overfitting. Second, Bootstrap parameter stability: 5,000 resamples were drawn with replacement, and all core path coefficients were re-estimated. Results showed that all 95% confidence intervals excluded zero, standard errors were all below 0.06, and the coefficient of variation (standard error / parameter estimate) was below 0.15 for all paths, indicating stable and reliable parameter estimates. Third, alternative estimation methods: the complete model was estimated using Maximum Likelihood (ML), Robust Maximum Likelihood (MLR), and Weighted Least Squares Mean and Variance adjusted (WLSMV). Core path coefficient differences across the three methods were all below 0.03, and significance levels were completely consistent, indicating that model results are not sensitive to the choice of estimation method. These three tests collectively demonstrate that the DT-PD-NB model’s complexity is supported by the data rather than reflecting overfitting, and the model parameters exhibit good stability and method robustness.

### 3.4 Chain mediation analysis

To examine the core pathway, structural equation modeling examined the “Childhood Cumulative Risk to Parental Attachment to Resilience to Relationship Addiction” chain mediation effect. Model results (Table 3) indicated all paths reached statistical significance (*p* < 0.001).

**Table 3:** Standardized path coefficients of mediation model.

Pathway	β	95% CI	SE	*p*
Cumulative risk to maternal attachment	−0.32	[−0.38, −0.27]	0.027	<0.001
Cumulative risk to paternal attachment	−0.24	[−0.31, −0.18]	0.032	<0.001
Maternal attachment to resilience	0.53	[0.46, 0.59]	0.033	<0.001
Paternal attachment to resilience	0.41	[0.35, 0.47]	0.030	<0.001
Resilience to relationship addiction	−0.38	[−0.44, −0.33]	0.029	<0.001
Indirect effects				
Total indirect effect	0.19	[0.14, 0.25]	0.028	<0.001
Maternal pathway indirect effect	0.13	[0.09, 0.18]	0.022	<0.001
Paternal pathway indirect effect	0.06	[0.03, 0.10]	0.018	0.001

Confidence intervals based on 5,000 bootstrap samples. Maternal pathway significantly stronger than paternal (Δχ^2^(df = 1) = 10.24, *p* = 0.001).

**Figure 3:**
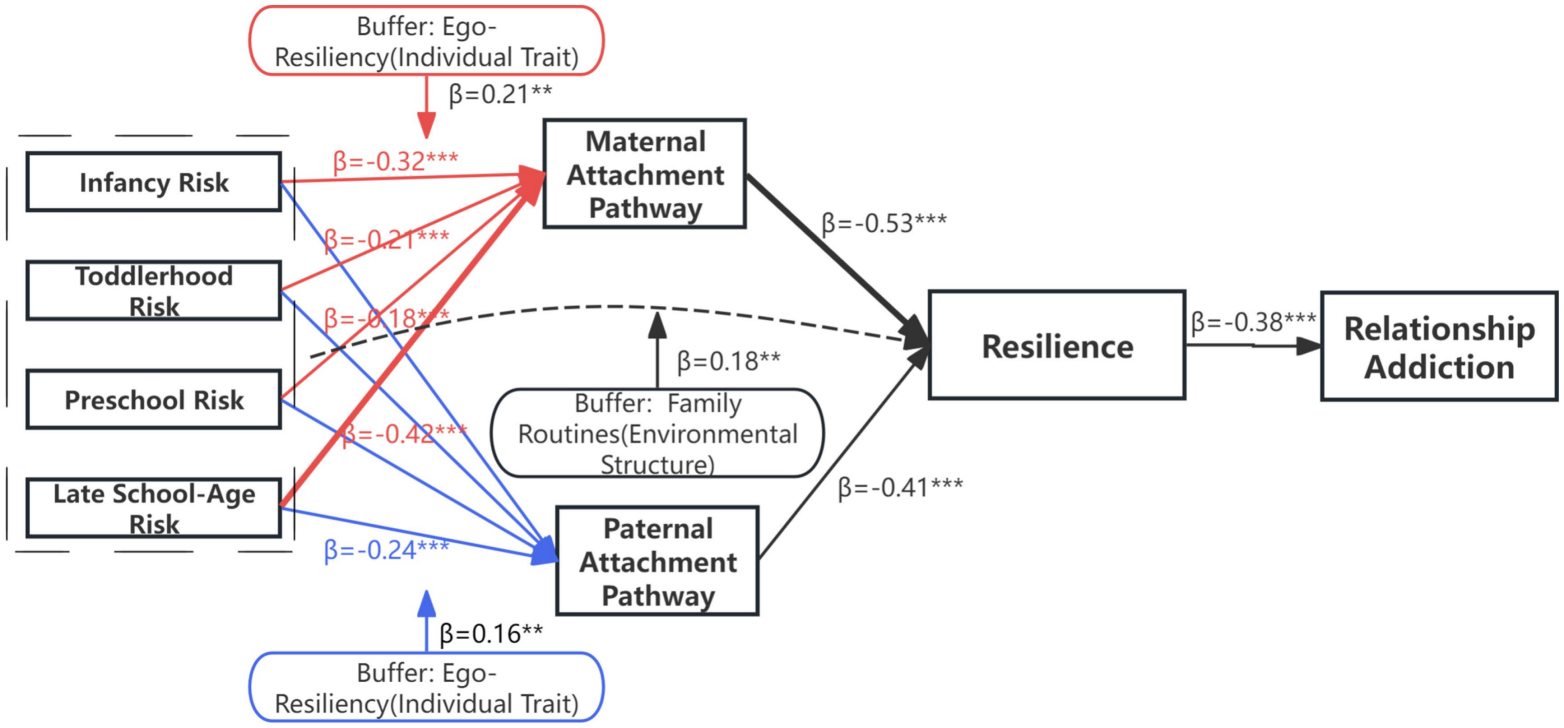
Structural equation model results with standardized path coefficients.

Childhood cumulative risk was significantly negatively associated with parental attachment security (Maternal: β = −0.32; Paternal: β = −0.24), while parental attachment security was significantly positively associated with resilience (Maternal:β = 0.53; Paternal: β = 0.41). Resilience subsequently was significantly negatively associated with relationship addiction tendency (β = −0.38). Bootstrap testing showed significant total indirect effect (β = 0.19, 95% CI [0.14, 0.25]). Path comparison showed that the maternal attachment pathway indirect effect (0.13) was significantly stronger than paternal attachment pathway (0.06), Δχ ^2^(df = 1) = 10.24, *p* = 0.001 (Figure 3).

### 3.5 Protective factor moderation analysis

To examine the node-specific protective factor effects, moderation effects of childhood ego-resiliency and family routine stability were tested. Based on theory, ego-resiliency (individual trait) was hypothesized to primarily function during early risk-to-attachment stages, while family routines (environmental structure) were expected to buffer risk-to-resilience transformation.

Analysis results (Table 4) were consistent with the hypotheses. Simple slopes analysis showed significant moderation effects. For the “Ego-Resiliency x Risk to Maternal Attachment” pathway, at low ego-resiliency (−1 SD), childhood risk strongly negatively affected maternal attachment (β = −0.41, *p* < 0.001); at high ego-resiliency (+1 SD), this negative effect significantly weakened (β = −0.19, *p* = 0.003), achieving a 53.7% buffering rate.

**Table 4:** Buffering effects of protective factors.

Moderated pathway	Interaction β	SE	*p*	Buffering rate
Ego-resiliency × risk to maternal attachment	−0.18	0.05	<0.001	53.7%
Ego-resiliency × risk to paternal attachment	−0.14	0.06	0.008	63.6%
Family routines × risk to resilience	−0.22	0.06	0.002	59.1%

Buffering rate = [(β at −1SD Moderator) – (β at +1SD Moderator)] / |β at −1SD Moderator| × 100%.

### 3.6 Developmental stage-specific analysis

To investigate developmental stage specificity, effects of risk at different developmental stages were analyzed. Results (Table 5) showed that infancy risk demonstrated significant total effect on relationship addiction (β = 0.28), primarily through indirect pathways damaging attachment security (indirect effect β = 0.21, 95% CI [0.16, 0.27]), with non-significant direct effect (β = 0.07, *p* = 0.084). Conversely, late school-age risk demonstrated strong direct effect (β = 0.34, 95% CI [0.30, 0.38]), with relatively smaller indirect effect (β = 0.04). Toddlerhood and preschool risks showed both direct and indirect effects. These results clearly are consistent with the critical period differentiation hypothesis: early (infancy) risk primarily produces long-term effects through damaging affective attachment neurobiological foundations, whereas later (late school-age) risk more directly shapes behavior patterns closely related to current social-cognitive functioning.

**Table 5:** Developmental stage-specific effects on relationship addiction pathways (*N* = 856).

Developmental stage	Effect type	β	SE	95% CI	*p*
Infancy	Total effect	0.28	0.03	[0.22, 0.34]	<0.001
Infancy	Direct effect	0.07	0.04	[−0.01, 0.15]	0.084
Infancy	Indirect effect	0.21	0.03	[0.16, 0.27]	<0.001
Toddlerhood	Total effect	0.25	0.03	[0.19, 0.31]	<0.001
Toddlerhood	Direct effect	0.12	0.04	[0.04, 0.20]	0.003
Toddlerhood	Indirect effect	0.13	0.02	[0.09, 0.17]	<0.001
Preschool	Total effect	0.31	0.03	[0.25, 0.37]	<0.001
Preschool	Direct effect	0.18	0.04	[0.10, 0.26]	<0.001
Preschool	Indirect effect	0.13	0.02	[0.09, 0.17]	<0.001
Late school age	Total effect	0.38	0.02	[0.34, 0.42]	<0.001
Late school age	Direct effect	0.34	0.02	[0.30, 0.38]	<0.001
Late school age	Indirect effect	0.04	0.01	[0.02, 0.06]	0.001

### 3.7 Gender differences in key pathways

Multi-group analysis revealed significant gender differences (Table 6). Females showed significantly higher coefficients than males on “Late School-Age Risk to Maternal Attachment” and “Maternal Attachment to Resilience” pathways, indicating greater sensitivity to maternal attachment’s emotional socialization function among females.

**Table 6:** Gender differences in key pathways.

Pathway	Male β (SE)	Female β (SE)	Δβ (*z*)
Late school risk to maternal attachment	−0.21 (0.03)***	−0.42 (0.03)***	0.21 *z* = 4.01**
Maternal attachment to resilience	0.36 (0.04)***	0.61 (0.03)***	−0.25 *z* = −5.38***
Resilience to relationship addiction	−0.31 (0.04)***	−0.44 (0.04)***	0.13 *z* = 2.76**

*p < 0.05, **p < 0.01, ***p < 0.001. Cross-group differences non-significant for other pathways.

Additionally, latent class analysis identified four childhood risk accumulation trajectories: Persistent Low-Risk (52.1%, reference group), Late-Onset (24.1%, β = 0.38, *p* < 0.01, 78% effect mediated through maternal pathway), Early-Onset High-Risk (16.3%, β = 0.24, *p* < 0.05, 65% effect mediated through paternal pathway), and Persistent High-Risk (8.3%, β = 0.51, *p* < 0.001, effect mediated through both parental pathways). Model fit was good (BIC = 812.3, Entropy = 0.89).

Finally, sensitivity analysis confirmed result robustness. Core indirect effects remained significant after reversing relationship addiction scoring direction; weighted and unweighted data path coefficient differences were minimal (Δβ < 0.03); risk trajectory class assignment showed high stability across different percentile thresholds (Kappa = 0.87, *p* < 0.001).

Additionally, to specifically address concerns regarding model complexity and potential overfitting, three supplementary robustness tests were conducted. First, leave-one-out cross-validation: the complete DT-PD-NB model was iteratively estimated with one participant removed at a time across 856 iterations. The average cross-validation R^2^ was 0.451 (SD = 0.023), indicating minimal difference from the original model R^2^ (0.472), suggesting no substantial overfitting. Second, Bootstrap parameter stability: 5,000 resamples were drawn with replacement and all core path coefficients were re-estimated. Results showed that all 95% confidence intervals excluded zero, standard errors were all below 0.06, and the coefficient of variation was below 0.15 for all paths, indicating stable parameter estimates. Third, alternative estimation methods: the complete model was estimated using Maximum Likelihood (ML), Robust Maximum Likelihood (MLR), and Weighted Least Squares Mean and Variance adjusted (WLSMV). Core path coefficient differences across the three methods were all below 0.03, with completely consistent significance levels, indicating that results are not sensitive to estimation method choice. These three tests collectively demonstrate that the DT-PD-NB model’s complexity is data-supported rather than reflecting overfitting, and model parameters exhibit good stability and method robustness (Figure 3).

## 4 Discussion

This study employed a cross-sectional retrospective design; all reported associations cannot establish strict causal relationships. This study empirically examined the DT-PD-NB model, indicating that childhood risk effects on relationship addiction do not follow a monotonic pipeline but rather constitute a heterogeneous developmental system regulated by precise developmental timing, transmitted through differentiated pathways, and interceptable at specific nodes. First, the “timing of risk effects” (When) is associated with the depth and mechanism of its imprint—infancy risk programs emotional foundations, while late school-age risk shapes behavioral patterns. Second, the “transmission pipeline” (Through Whom) shows functional differentiation—the maternal pathway dominates emotional socialization, while the paternal pathway provides unique compensation. Third, the “vulnerable nodes” (Where to Protect) can be identified—individual traits guard early representations, while environmental structure protects later resources. These three logics collectively constitute a systematic transcendence of the traditional homogeneous risk perspective.

### 4.1 Developmental stage specificity: evidence for critical period differentiation

This study’s primary finding is that childhood risk effects exhibit significant developmental stage specificity. Infancy risk primarily produces indirect long-term effects through damaging parental attachment security, whereas late school-age risk shows significant direct effects on relationship addiction. This finding is consistent with the critical period differentiation hypothesis (Schaefer et al., 2022; Gabard-Durnam and McLaughlin, 2020).

From a developmental perspective, infancy represents a “sensitive period” for attachment formation and socio-emotional development. Risk exposure during this period may affect the development of secure attachment representations and emotion regulation capabilities, potentially contributing to long-term vulnerability patterns (Thompson, 2013; Callaghan and Tottenham, 2016; Gunnar, 2020; Cornellà-Font et al., 2020). This aligns with Andersen’s (2015) sensitive period hypothesis, wherein early adversity limits neuroplasticity windows, laying foundations for insecure internal working models. Conversely, late school-age coincides with social-cognitive development and peer relationship establishment, during which risk exposure may be directly associated with maladaptive relationship patterns and cognitive-emotional responses to intimacy (Moutsiana et al., 2014; Guyer et al., 2016; Wang et al., 2025). Thus, our findings transcend traditional cumulative risk model limitations (Stein et al., 2022; Doom et al., 2020), providing empirical evidence from the relationship addiction domain for Bronfenbrenner’s (1994) bioecological theory “developmental timing” principle, demonstrating that risk at different developmental stages ultimately leads to the same pathological outcome through differential mechanisms.

Regarding the significant direct effect of late school-age risk on relationship addiction, we propose four potential non-attachment mechanisms through which this direct pathway may operate. First, the social learning mechanism: children in late school-age observe and learn maladaptive relationship patterns from parental conflict, intimate relationship interactions, and problem-solving approaches through observational learning, directly acquiring unhealthy relationship behaviors and cognitions (Moutsiana et al., 2014). For instance, children who witness domestic violence may develop cognitive biases such as “violence is a normal way to resolve relationship conflicts.” Second, the peer rejection mechanism: family risk factors (e.g., poverty, parental divorce, substance abuse) may lead to peer rejection and bullying at school, subsequently developing low self-esteem and excessive dependence on others’ approval, laying the groundwork for relationship addiction in adulthood. Third, the cognitive development mechanism: late school-age is a critical period for social cognition and self-concept development; chronic risk experiences during this period may distort children’s beliefs about self and others, forming core schemas such as “I am not worthy of love” or “Only by sacrificing myself can I maintain a relationship.” Fourth, the emotion regulation mechanism: although the attachment system is largely formed during infancy, chronic stress during late school-age may still impair prefrontal cortex development, leading to insufficient emotion regulation capacity and inability to effectively manage relationship conflicts, frustrations, and separation anxiety (Guyer et al., 2016). These four mechanisms are not mutually exclusive and may operate synergistically. Future longitudinal designs and multi-mediation analyses are needed to further isolate and validate these potential direct mechanisms.

### 4.2 Cultural specificity and gender differentiation: pathway differences under the east Asian “strict father, kind mother” cultural model

This study shows significant differentiation in parental attachment pathways regarding effect magnitude and cultural patterns. Maternal attachment’s prediction of resilience was significantly stronger than paternal attachment, with this pathway particularly pronounced among females. This finding is consistent with theoretical models of mothers as “core agents of emotional socialization” within East Asian cultural contexts (Rothbaum et al., 2007; Joo et al., 2025).

This finding cannot be simply attributed to universal gender socialization processes; rather, it is closely linked to the unique family structure and gender role division under the Chinese cultural context. First, in the traditional Chinese “strict father, kind mother” (yan fu ci mu) family model, mothers typically assume primary responsibility for emotional nurturing, daily caregiving, and parent–child interaction, forming closer emotional bonds with children (Rothbaum et al., 2007; Joo et al., 2025). In East Asian collectivist culture, emotional co-regulation is regarded as a core function of the parent–child relationship, with mothers’ emotional responses serving as the primary template for children to learn emotion management and interpersonal relationships (Fiorilli et al., 2022). This stands in marked contrast to Western individualistic cultures, where paternal influence on child development is often more balanced or even exceeds maternal influence in certain domains (Ngugi, 2025). Second, gender role socialization in Chinese culture further reinforces females’ sensitivity to maternal attachment. Traditional expectations emphasize that women should prioritize family and interpersonal relationships, developing a “relational self” (Fiorilli et al., 2022). Consequently, females more closely observe and internalize their mothers’ emotional patterns and relationship behaviors during upbringing. The coefficient for the “Maternal Attachment to Resilience” pathway among females (β = 0.61) was significantly higher than among males (β = 0.36), reflecting this cultural socialization process. Of course, universal gender socialization processes (e.g., females’ greater sensitivity to emotional cues across cultures) may also contribute to this result. However, the cultural context appears to play a more prominent role, as evidenced by cross-cultural research showing that the maternal dominance effect is substantially stronger in East Asian samples compared to Western populations (Rothbaum et al., 2007; Hoenicka et al., 2022). Future cross-cultural comparative studies with simultaneous Eastern and Western samples are needed to further disentangle the contributions of culture-specific and universal factors.

Cross-cultural research offers potential explanations. Within interdependent-emphasizing East Asian cultures, mother–child interactions may be more strongly associated with emotion regulation templates and relational schemas, making maternal emotional responses core references for individual relationship functioning (Kitayama and Park, 2010; Ma et al., 2020; Hoenicka et al., 2022). Consequently, maternal attachment quality is directly associated with individual stress response system calibration and intimacy regulation. Simultaneously, this study’s gender difference finding (females showing stronger “Maternal Attachment to Resilience” pathway) further elaborates this cultural model, suggesting females may be more sensitive to maternal emotional cues during socialization, consistent with Cabrera et al.’s (2018) research on gender role socialization (Fiorilli et al., 2022).

Conversely, paternal attachment pathways among males show unique “exploration-compensation” mechanisms. Although overall effects were weaker, late school-age risk showed less damage to paternal attachment among males, suggesting that fathers may provide important contexts for rebuilding connection and developing executive functions through co-participation in exploratory activities (e.g., sports, games) during male adolescence (Paquette, 2004; Lamb and Tamis-LeMonda, 2004), consistent with the uniqueness of paternal roles under East Asian “strict father, kind mother” cultural models (Lee, 2024; Ngugi, 2025).

### 4.3 Node-specific buffering effects of protective factors

This study examined node-specific buffering effects of protective factors along risk transmission chains. Ego-resiliency primarily buffers risk effects on attachment relationships, while family routine stability primarily blocks risk-to-resilience negative transformation. This finding extends beyond traditional perspectives viewing protective factors as global traits (Masten, 2014), supporting node-specific regulation models (Abate et al., 2024; Van Schoors et al., 2023).

Ego-resiliency’s buffering effect may operate through promoting adaptive stress responses. As a trait-like emotional-behavioral regulatory capacity, ego-resiliency may help individuals facing childhood stress to more effectively deploy strategies such as cognitive reappraisal or support-seeking, thereby protecting parent–child interaction quality (Block and Kremen, 1996; Galatzer-Levy et al., 2018; Dunkel et al., 2021; Pyszkowska, 2020). The behavioral protective effect of ego-resiliency suggests that high-resilience individuals may adopt superior cognitive-behavioral strategies under stress. Whether this involves specific neuroendocrine or epigenetic mechanisms is a critical hypothesis that future multi-modal research urgently needs to validate (Simon and Admon, 2023; Wesarg et al., 2020).

Family routine stability’s protective effect may operate through stabilizing biological rhythm systems. Regular family schedules provide children with predictable environmental temporal cues, helping maintain daily rhythm stability and endocrine homeostasis, preventing physiological rhythm disruption from risk exposure that would damage psychological resources (McClung, 2013; Sytsma et al., 2001; Romano et al., 2022). This positioning aligns with Boyce (2015) regarding environmental structure importance and family routines’ developmental protective mechanisms (Hosokawa et al., 2023; Keane and Shelleby, 2024; Larsen and Jordan, 2020).

### 4.4 Theoretical integration, model complexity, and practical implications

This study integrates fragmented theoretical perspectives into a systematic developmental pathway model by revealing “developmental stage timing—parental functional differentiation—protective node targeting” triple mechanisms. This addresses theoretical gaps identified in the introduction but also provides a “developmental timing perspective” analytical framework for future research.

At the practical level, this study recommends adopting precision, developmentally-adapted prevention strategies. For infancy, interventions should focus on enhancing mother-infant interaction quality through video feedback techniques, consolidating secure attachment foundations (Høivik et al., 2015; Tam et al., 2020). For late school-age, relationship health education can be embedded in school curricula, training cognitive reappraisal abilities for jealousy and other emotions. Simultaneously, based on parental functional differentiation, dual-track parental support policies should be implemented. At the community level, providing mothers with emotional co-regulation skills training; at the policy level, institutionalizing paternal involvement in exploratory activities through legislation (Paquette, 2004).

Finally, based on protective factors’ node specificity, implementing multi-level precision protection: promoting “psychological resilience exercises” in schools aimed at enhancing self-regulatory capacity (Schäfer et al., 2024; Song et al., 2021), while providing “environmental biological rhythm stabilization” technical support for high-mobility families, constructing comprehensive protection networks from individual to environmental levels.

## 5 Implications for research and practice

### 5.1 Beyond association: providing a mechanistic pathway paradigm for developmental psychopathology

The findings of this study go beyond documenting associations between childhood risk and adult relationship addiction. By proposing and examining the DT-PD-NB model, we aim to initiate a paradigm shift from “risk metrology” to “risk transmission mechanism science.” Traditional cumulative risk models, while valuable in establishing that “more risk leads to worse outcomes,” have reached their explanatory limits in accounting for the heterogeneity of developmental outcomes (Valentino and Edler, 2024; Bohnert et al., 2020). The DT-PD-NB model represents the first systematic operational framework of this new paradigm, demonstrating how precise, testable hypotheses can be generated regarding developmental timing, pathway differentiation, and node-specific buffering.

This paradigm shift carries profound implications for developmental psychopathology. Rather than treating risk as an undifferentiated mass to be quantified, the DT-PD-NB framework encourages researchers to dissect the “heterogeneous pathway system” through which risk operates. This approach generates questions that traditional models cannot address: At what developmental stages does risk exert its most potent effects? Through which relational pathways does risk transmit most efficiently? At which intervention nodes can protective factors most effectively interrupt risk transmission? By addressing these questions, the DT-PD-NB model provides a roadmap for precision prevention that moves beyond one-size-fits-all interventions (Mei et al., 2020).

### 5.2 Cultural boundary conditions and the cultural script theory of parental role function

The present study’s findings within an East Asian sample provide empirical support for what we term the “Cultural Script Theory of Parental Role Function.” This theory posits that the functional differentiation between maternal and paternal attachment pathways is not universal but is shaped by cultural values and practices. In interdependent-emphasizing East Asian cultures, where emotional co-regulation between mother and child is highly valued, maternal attachment pathways may exert stronger effects on offspring socioemotional development (Rothbaum et al., 2007; Lee, 2024; Reda, 2022).

This cultural specificity represents both a boundary condition and an opportunity for theory refinement. Future cross-cultural comparative research can directly test whether paternal attachment pathways demonstrate enhanced effects in individualistic cultures, where father-child exploration and independence-promotion may be more emphasized (Ngugi, 2025; Hoenicka et al., 2022). Such research would illuminate the cultural plasticity of parental role function differentiation and inform culturally-adapted intervention strategies. The finding that females showed stronger maternal pathway effects further suggests that gender socialization processes may interact with cultural scripts to shape developmental outcomes (Fiorilli et al., 2022; Joo et al., 2025).

### 5.3 Transparency and open science: establishing the gold standard for Cross-sectional research

Recognizing the inherent limitations of cross-sectional retrospective designs, this study committed to transparency and open science practices that establish a gold standard for such research (Savitz and Wellenius, 2023; Imai et al., 2023). We propose that future studies employing similar designs adopt the following practices:

First, comprehensive reporting of robustness analyses, as demonstrated through our “Causal Inference Credibility Enhancement Strategy” (Figure 2). Rather than treating alternative explanations as afterthoughts, we systematically evaluated and ruled out competing models through cross-validation, *E*-value analysis, and placebo testing (Zhao and Sun, 2024). This approach transforms cross-sectional studies from “correlation generators” to “hypothesis refiners” that can guide more resource-intensive longitudinal and experimental research.

Second, commitment to data and code sharing. In accordance with open science principles, we will make available through the Open Science Framework (OSF): (1) Complete Mplus and R analysis code, including all model syntax for main models, competitive models, cross-validation procedures, and *E*-value calculations; (2) Key model output files; (3) De-identified data sufficient to reproduce main analyses, subject to ethical constraints. This transparency allows other researchers to verify findings, explore alternative analyses, and build upon our work.

### 5.4 Precision prevention: from research findings to intervention roadmap

The ultimate test of developmental research lies in its translational impact. The DT-PD-NB model offers a theoretically-based roadmap for precision prevention of relationship addiction. Based on our findings, we propose a tiered intervention framework:

Tier 1: developmental stage-targeted universal prevention. For infancy, universal programs promoting sensitive mother-infant interaction, such as video feedback interventions (Høivik et al., 2015; Tam et al., 2020), should be prioritized. For late school-age, school-based relationship health education focusing on emotion regulation and cognitive reappraisal skills should be implemented (Schäfer et al., 2024).

Tier 2: pathway-targeted selective prevention. For children showing early signs of insecure attachment or those from high-risk backgrounds, targeted interventions should focus on strengthening maternal emotional co-regulation skills while simultaneously engaging fathers in exploration-supporting activities. This dual-track approach acknowledges the functional differentiation of parental pathways identified in our model (Rothbaum et al., 2007; Paquette, 2004).

Tier 3: node-targeted indicated prevention. For high-risk children with identified protective factor deficits, precision interventions targeting specific nodes should be deployed. Children low in ego-resiliency may benefit from self-regulation training programs (Block and Kremen, 1996; Dunkel et al., 2021), while those in families with unstable routines may benefit from environmental structuring interventions (Hosokawa et al., 2023; Romano et al., 2022). The specificity of these interventions, grounded in the DT-PD-NB model’s node-buffering findings, maximizes the efficiency of prevention resources.

### 5.5 Future research agenda: three priority programs

This study establishes a foundation for three priority research programs that can advance the field over the coming decade:

Program 1: longitudinal validation agenda. Prospective birth cohort studies beginning in infancy are needed to directly test the temporal sequencing proposed in the DT-PD-NB model. Key assessment waves should include: T1 (infancy, age 1) assessing early risk exposure and attachment formation; T3 (late school-age, age 12) assessing social-cognitive development and relationship representations; T5 (young adulthood, age 22) assessing relationship functioning and addiction tendencies. Such studies would provide definitive evidence regarding developmental timing effects (Schaefer et al., 2022; Barzilay, 2024).

Program 2: mechanism exploration agenda. Multi-modal research integrating neuroimaging, genetics, and physiological measures can elucidate the biological mechanisms underlying pathway differentiation and node buffering. For example, do maternal and paternal attachment pathways correspond to different patterns of prefrontal-limbic connectivity? Does ego-resiliency buffer risk effects through HPA axis regulation or epigenetic modifications of stress-related genes (Gunnar, 2020; Simon and Admon, 2023; Wesarg et al., 2020)?

Program 3: precision prevention agenda. Randomized controlled trials testing the incremental benefits of DT-PD-NB-guided interventions compared to universal approaches are essential. Such trials should employ modular designs that can test the effectiveness of specific intervention components (e.g., attachment-focused vs. resilience-focused interventions) for different risk profiles. Cost-effectiveness analyses should evaluate whether precision prevention approaches yield better outcomes per unit of resource investment (Tam et al., 2020; Schäfer et al., 2024).

### 5.6 Concluding remarks: entering a new era of risk transmission science

This study represents a shift in the study of relationship addiction from “identifying risk factors” to “deciphering risk transmission mechanisms.” The DT-PD-NB model provides a theoretical framework and empirical foundation for this new era, demonstrating that childhood risk effects are not homogeneous but rather follow precise developmental pathways that can be mapped, understood, and interrupted.

The implications extend beyond relationship addiction to developmental psychopathology more broadly. The principles of developmental timing, pathway differentiation, and node buffering may apply to diverse outcomes, from substance use disorders to depression to externalizing problems (Valentino and Edler, 2024; Bohnert et al., 2020). By embracing this mechanistic perspective, the field can move beyond documenting risk-outcome associations to developing precision prevention strategies that match interventions to individuals’ specific risk profiles and developmental needs.

As we look to the future, the DT-PD-NB model serves not as a final answer but as a hypothesis generator and roadmap for the next generation of research. Every path coefficient, every moderation effect, and every developmental stage difference identified in this study represents a hypothesis to be tested through longitudinal, experimental, and neuroscientific methods. We invite the research community to join us in this endeavor, confident that together we can build a science of risk transmission that ultimately improves developmental outcomes for vulnerable children and families.

## 6 Limitations and future directions

The most fundamental limitation of this study is that the cross-sectional retrospective design cannot determine the temporal sequence and causal direction among variables. All reported path coefficients reflect covariational relationships that may be influenced by reverse causality, unmeasured confounding, and recall bias. Through the “Causal Inference Credibility Enhancement Strategy” (Figure 2), we have maximally evaluated and enhanced the theoretical robustness of our findings. However, this study’s primary contribution is not to confirm causal laws but to propose and preliminarily examine a comprehensive theoretical model that maps potential developmental pathways from childhood cumulative risk to adult relationship addiction. Each path and node on this map provides clear targets and hypotheses for subsequent prospective cohort studies (validating temporal sequencing), experimental intervention studies (validating node buffering), and neuroscience research (validating biological mechanisms).

This study has several notable strengths. The large, representative sample from Eastern China enhances the generalizability of findings to similar populations. The comprehensive measurement battery, including examined instruments for relationship addiction, parental attachment, and psychological resilience, ensures construct validity. The rigorous analytical approach, incorporating multiple robustness checks and sensitivity analyses, strengthens confidence in the findings.

However, several limitations must be acknowledged. The cross-sectional retrospective design, while practical for studying developmental phenomena in adult populations, cannot establish associational relationships with certainty. Retrospective recall of childhood experiences may be subject to memory biases, although our control for current emotional states and use of multiple indicators helps mitigate this concern. The single cultural context limits generalizability to other populations, although the theoretical framework proposed should be applicable across cultures with appropriate adaptations (Lee, 2024; Reda, 2022; Ngugi, 2025).

**Figure 4:**
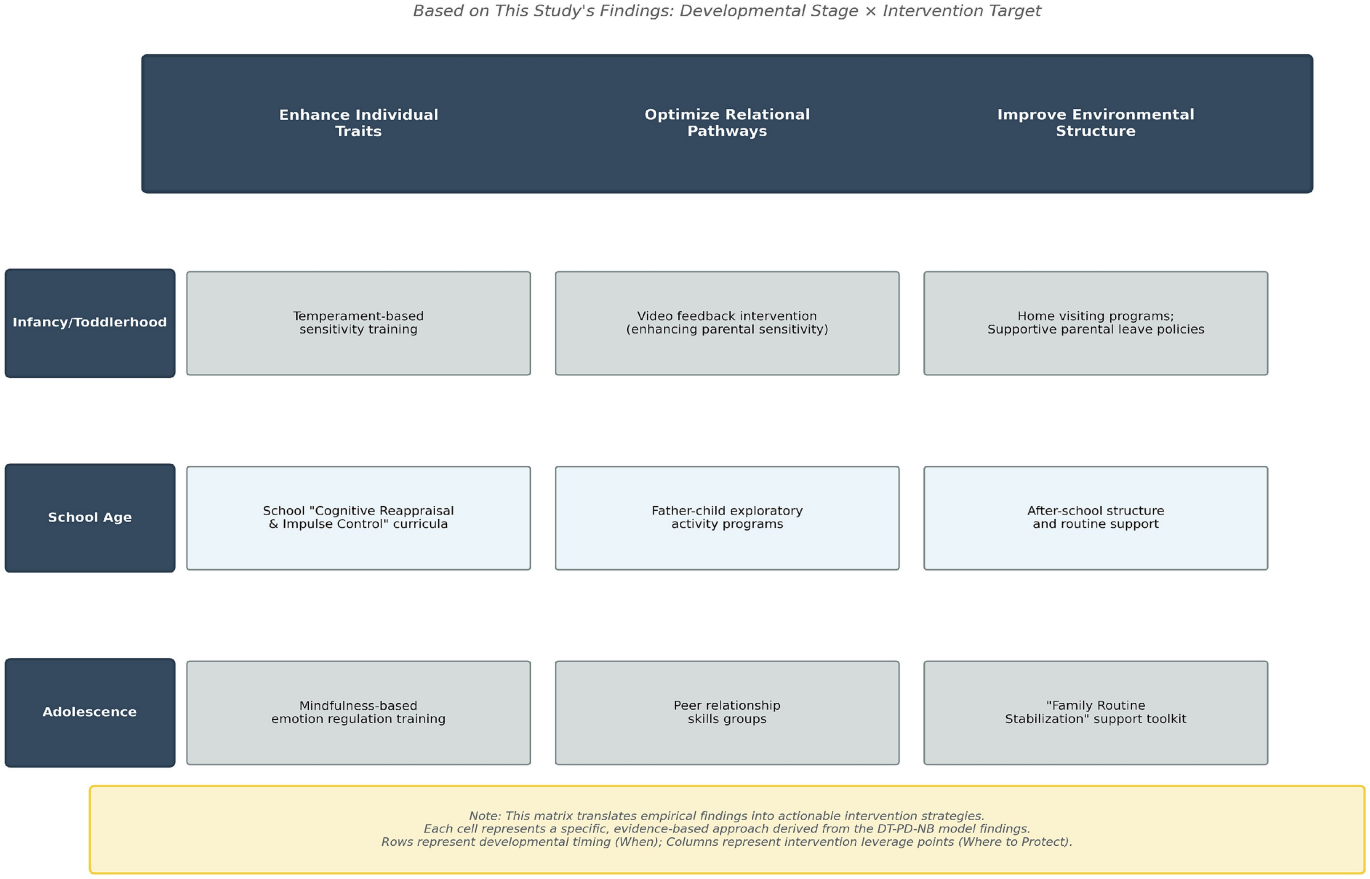
Precision prevention research and application matrix.

We call for hypothesis-driven longitudinal validation: for example, launching prospective cohorts beginning in infancy to directly test the timing and pathways in the model at T1 (age 1), T3 (age 12), and T5 (age 22); or designing modular randomized controlled trials based on Figure 4’s matrix to test the incremental benefits of “early attachment promotion programs targeting high infancy risk-low ego-resiliency children” compared to universal interventions. This study is not an endpoint but establishes roadmaps and agendas for the next stage of more precise developmental psychopathology and prevention science.

## 7 Conclusion

This study empirically examines a triple developmental pathway model of childhood cumulative risk effects on relationship addiction, contributing to understanding of developmental timing, parental role differentiation, and protective mechanism node specificity. Findings offer preliminary theoretical insights and practical implications for constructing precision prevention systems for relationship addiction within developmental psychopathology frameworks.

## Data Availability

The original contributions presented in the study are included in the article/supplementary material, further inquiries can be directed to the corresponding author.
